# Acrodermatitis enteropathica in the pediatric population: a literature review of real-world studies

**DOI:** 10.3389/fnut.2025.1590075

**Published:** 2025-06-13

**Authors:** Wanlin Cui, Ningning Wang, Mingyue Shi, Xuegang Xu, Hongkun Jiang

**Affiliations:** ^1^Department of Pediatrics, The First Hospital of China Medical University, Shenyang, Liaoning, China; ^2^Department of Dermatology, The First Hospital of China Medical University, Shenyang, Liaoning, China; ^3^NHC Key Laboratory of Immunodermatology, National Joint Engineering Research Center for Theranostics of Immunological Skin Diseases, China Medical University, Shenyang, Liaoning, China

**Keywords:** acrodermatitis enteropathica, zinc deficiency, *SLC39A4*, metabolic disorder, pediatric dermatology

## Abstract

Acrodermatitis enteropathica (AE) primarily affects children and is characterized by periorificial and acral dermatitis, alopecia, and diarrhea. Currently, zinc remains an effective treatment for pediatric AE. However, there is a lack of consensus regarding the optimal dosage for zinc therapy. Furthermore, emerging evidence suggests that basic zinc supplementation may be ineffective in certain cases. Therefore, we performed a literature search using PubMed and Web of Science in July 2024, focusing on pediatric AE patients, and reviewed 190 articles involving 231 patients (aged < 18 years) to address the gaps in knowledge about hereditary and acquired zinc deficiencies in these patients and to evaluate cases occurring during metabolic disease decompensation. In summary, zinc deficiency was observed in 75.9% of the patients. Among the 174 AE patients who received zinc supplementation at various dosages, 159 (91.4%) demonstrated therapeutic efficacy, with 1–3 mg/kg/day as the most commonly used effective dosage. Additionally, zinc supplementation was frequently shown to be ineffective in patients with AE associated with metabolic disorders. It is imperative to address the underlying metabolic perturbations to achieve optimal management of this condition. Therefore, a comprehensive evaluation of pediatric patients is crucial when addressing cases of AE.

## Introduction

Acrodermatitis enteropathica (AE) is a rare condition characterized by periorificial and acral dermatitis, alopecia, and diarrhea; and was first described by Danbolt and Closs in 1942 (1). Historically, AE was considered an autosomal recessive disorder (2). With the discovery of zinc supplementation as a treatment for AE in approximately 1974 (3), acquired zinc deficiency was incorporated into the etiology of AE (4, 5). Since 2002, researchers believed that AE was linked to an inherited genetic defect in the *SLC39A4* gene affecting the zinc transporter *Zip4* (6). However, recent findings suggest that the link between metabolic disorders and AE may be more complex: several metabolic disorders have been reported to manifest as AE-like syndromes (7), some of which are unrelated to zinc deficiency (8, 9). This broader spectrum of underlying causes indicates that AE may not solely be attributed to zinc transport defects, highlighting the importance of considering metabolic disorders in the diagnosis and management of AE.

Currently, there are no systematic reviews that encompass all published cases of pediatric AE, including those related to hereditary and acquired zinc deficiencies, as well as cases arising from metabolic disorders. Moreover, although zinc supplementation remains the cornerstone of therapy for AE, there is currently a lack of consensus regarding the appropriate dosage for zinc treatment. While lifelong supplementation of elemental zinc at a dosage of 3 mg/kg/day is recommended, variations in practice persist (10). Therefore, we conducted a systematic review of the literature to gain a deeper understanding of all subtypes of pediatric AE, with a specific emphasis on their therapeutic approaches.

## Methods

We adhered to the Preferred Reporting Items for Systematic reviews and Meta-Analyses (PRISMA) guidelines for conducting and reporting this systematic review. We aimed to improve the diagnosis and treatment of AE in clinical practice.

### Search strategy and selection criteria

**Figure 1:**
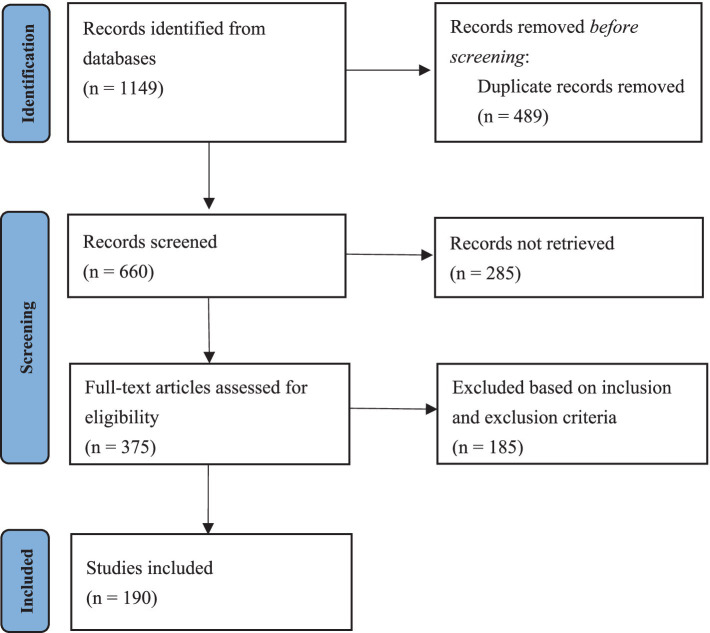
A flowchart summarizing the process of selecting patients with AE in the pediatric population based on the PRISMA guidelines.

We conducted PubMed and Web of Science database searches in July 2024 for all the literature on pediatric patients with AE using the following key words: “acrodermatitis enteropathica” AND “pediatric” OR “infant” OR “toddler” OR “child” OR “adolescent.” The review protocol was registered in the Open Science Framework (Registration DOI: https://doi.org/10.17605/OSF.IO/BN3TD). Studies from all years were considered. A flowchart summarizing the process of selecting studies on AE in the pediatric population is presented in Figure 1.

### Exclusion criteria

Studies were excluded according to the following criteria: age of onset over 18 years, review articles, or nondetailed cases.

### Data collection

The patient data recorded included gender, age of onset and diagnosis, cutaneous characteristics, lesion location, and initial lesion site. Other documented clinical manifestations included diarrhea, alopecia, ocular involvement, growth retardation, and neurological complications. Laboratory and dermatological tests were used to assess blood zinc levels, alkaline phosphatase (ALP) levels, albumin levels, hemoglobin levels, *SLC39A4* mutations, skin swab cultures, and biopsies. Additionally, medical history, treatment, and prognosis were recorded. Not all studies provided data on every measure, and the total number of patients included in each data analysis reflects the available records. Qualitative outcomes were analyzed descriptively, while quantitative outcomes were combined and analyzed using descriptive statistics. We performed the statistical analysis using SPSS 24.0 (IBM-SPSS, Chicago, USA). The chi-square test was applied in Tables 1, 2, with *χ*^2^ and *p* values calculated. A *p* value of less than 0.05 was considered statistically significant.

**Table 1:** Cross (Chi-square) analysis results of complications at different durations from onset to diagnosis.

Complications (*n*, %)^≠^	Time (w)		*χ*2	*p*
	< 4 (*n* = 34)	≥ 4 (*n* = 124)		
Neuropsychiatric manifestations	10 (29.41)	48 (38.71)	0.993	0.319
Growth retardation	2 (5.88)	38 (30.65)	8.654	0.003
Ocular abnormalities	1 (2.94)	11 (8.87)	0.625	0.429

^≠^Only patients with clearly defined onset and diagnosis times were analyzed.

**Table 2:** Cross (Chi-square) analysis results of the effects of zinc supplementation on lesion recovery.

Item, *n* (%)	Category	Dose (mg/kg/day)^#^				Total	*χ*2	*p*
		< 1	≥ 1 to ≤ 3	> 3 to ≤ 5	> 5			
Time (w)*	≤ 1	1 (3.57)	19 (67.86)	4 (14.29)	4 (14.29)	28	2.880	0.824
	> 1 to ≤ 4	2 (7.69)	18 (69.23)	4 (15.38)	2 (7.69)	26		
	> 4	0 (0.00)	8 (61.54)	2 (15.38)	3 (23.08)	13		
Total		3 (4.48)	45 (67.16)	10 (14.93)	9 (13.43)	67		

^#S^tudies that did not calculate zinc dosage based on body weight and those with unspecified zinc dosages were excluded from this table to enable a valid comparison between the different zinc dosage groups.

*Studies that did not provide information on the time to lesion recovery were excluded to allow for a clearer comparison of the effects of different zinc dosages on lesion recovery.

## Results

The initial literature search yielded 660 results after removing duplicates. Manual screening resulted in 190 studies, including a total of 231 patients (129 boys, 55.8%) (7, 8, 11–198). Among these studies, 189 were isolated case reports, and 1 was a retrospective study. The age at onset was recorded for 179 patients, with a mean age of 10.5 months (range: 0.2–188 months). Among the 216 patients, 189 (87.5%) experienced their first episode of AE, whereas 27 (12.5%) experienced recurrence. In 164 patients with defined onset and diagnosis times, the average duration from onset to diagnosis was 40 weeks (range: 0.3–1,008 weeks). Table 3 summarizes the detailed information.

**Table 3:** Summary of patient characteristics in this review.

Variable^+^	Patient total (*n* = 231)
Gender, *n* (%)	
Male	129 (55.8)
Female	102 (44.2)
Age at onset, m (range)	(*n* = 179) 10.5 (0.2–188)
Mode of onset, *n* (%)	(*n* = 216)
First episode	189 (87.5)
Recurrence	27 (12.5)
Clinical characteristic, *n* (%)	(*n* = 209)
Diarrhea	98 (46.9)
Alopecia	88 (42.1)
Neuropsychiatric manifestations	70 (33.5)
Irritability	62 (29.7)
Apathy or depression	8 (3.8)
Growth retardation	49 (23.4)
Fever	35 (16.8)
Ocular abnormalities	17 (8.1)
Photophobia	7 (3.4)
Blepharitis	6 (2.9)
Keratitis or conjunctivitis	5 (2.4)
Decreased vision with macular atrophy	1 (0.5)
Delayed puberty	2 (1.0)
Dermatologic manifestation, *n* (%)	
Lesion morphology	(n = 200)
Erythema	160 (80.0)
Desquamation	101 (50.5)
Blisters or vesicles	85 (42.5)
Erosion or ulceration	65 (32.5)
Pustules	22 (11.0)
Scaly macules	16 (8.0)
Exudation	11 (5.5)
Eczematous plaques	3 (1.5)
Pigmentation	2 (1.0)
Depigmentation	2 (1.0)
Location of skin lesions	(*n* = 225)
Hands and feet and limbs	187 (83.1)
Perioral area	171 (76.0)
Perianal area	163 (72.4)
Perineum	133 (59.1)
Cheeks	91 (40.4)
Eye corners	54 (24.0)
Trunk	50 (22.2)
Auricles	29 (12.9)
Occipital region	27 (12.0)
Location where the lesion first appeared	(n = 80)
Perianal area	28 (35.0)
Hands and feet and limbs	19 (23.8)
Perioral area	12 (15.0)
Cheeks	9 (11.3)
Perineum	7 (8.8)
Occipital region	2 (2.5)
Trunk	2 (2.5)
Auricles	1 (1.3)
Skin swab culture, *n* (%)	(*n* = 35)
Single bacterium	17 (48.6)
Mixed bacteria	8 (22.9)
Bacteria and fungi	6 (17.1)
Single fungus	4 (11.4)
Laboratory abnormalities, *n* (%)	
*SLC39A4* mutations	32
Zinc deficiency	145 (75.9) (n = 191)
Decreased ALP	40 (46.0) (n = 87)
Decreased albumin	33 (60.0) (n = 55)
Decreased hemoglobin	39 (61.9)(n = 63)
Causes of zinc deficiency, *n* (%)	(*n* = 63)
Insufficient zinc intake	37 (58.7)
Impaired absorption	38 (60.3)
Increased demand	10 (15.9)
Two or more contributing factors	13 (20.6)
Associated conditions, *n* (%)	(*n* = 231)
Metabolic diseases	57 (24.7)
CF	18 (31.6)
MSUD	15 (26.3)
MMA	7 (12.3)
OTC	4(7.0)
PA	4(7.0)
PKU	3 (5.3)
Biotinidase deficiency	1 (1.8)
Hartnup disease	1 (1.8)
D < 1.063 lipoproteins deficiency	1 (1.8)
Nonketotic hyperglycinemia	1 (1.8)
Aminoacidemia	1 (1.8)
Type 1 glutaric acidemia	1 (1.8)
Premature	28 (12.1)
Gastrointestinal diseases	20 (8.7)
Malnutrition	9 (3.9)
Treatment and prognosis, *n* (%)	
Treatment: zinc (mg/kg/day)	(n = 174)
< 1	4 (2.3)
≥ 1 to < 3	35 (20.1)
3	20 (11.5)
> 3 to ≤ 5	20 (11.5)
> 5	10 (5.8)
Not weight-adjusted	41 (23.6)
Unknown dosage	44 (25.3)
Prognosis: lesion recovery time (w)	
≤ 1	46 (26.4)
> 1 to ≤ 4	50 (28.7)
> 4	22 (12.6)
Unknown recovery time	41 (23.6)
Unknown outcome	5 (2.9)
Ineffective	10 (5.8)

^+^Data are presented as numbers (%) or means (ranges).

ALP, alkaline phosphatase; CF, cystic fibrosis; MSUD, maple syrup urine disease; MMA, methylmalonic acidemia; OTC, ornithine transcarbamylase deficiency; PA, propionic acidemia; PKU, phenylketonuria.

### Clinical characteristics

Among the 231 pediatric patients included, all exhibited dermatitis, with 209 having additional clinical symptoms recorded. Diarrhea was present in 98 patients (46.9%), and alopecia was present in 88 patients (42.1%). Only 44 patients (21.1%) exhibited the triad of dermatitis, diarrhea, and alopecia simultaneously. Other symptoms included neuropsychiatric manifestations in 70 patients (33.5%), irritability in 62 patients (29.7%), and apathy or depression in 8 patients (3.8%). Growth retardation was noted in 49 patients (23.4%), fever in 35 patients (16.8%), and ocular abnormalities in 17 patients (8.1%). Ocular issues included photophobia in 7 patients (3.4%), blepharitis in 6 patients (2.9%), keratitis or conjunctivitis in 5 patients (2.4%), and decreased vision with macular atrophy in 1 patient (0.5%). Delayed puberty was observed in 2 patients (1.0%). Notably, 28 patients (13.4%) had dermatitis as the sole manifestation of AE. The incidence of clinical manifestations—neuropsychiatric manifestations, growth retardation, and ocular abnormalities—was compared between patients whose time from onset to diagnosis was ≥ 4 weeks and patients whose time from onset to diagnosis was < 4 weeks. Table 1 shows that a delayed diagnosis was associated with an increased risk of growth retardation (*χ*^2^ = 8.654, *p* = 0.003).

### Dermatologic manifestations

Dermatitis can present in various forms among patients. A detailed analysis of the lesion morphology in 200 patients revealed that the most common lesion morphology was erythema, which was observed in 160 patients (80.0%). This was followed by desquamation in 101 patients (50.5%), blisters or vesicles in 85 patients (42.5%), erosion or ulceration in 65 patients (32.5%), pustules in 22 patients (11.0%), scaly macules in 16 patients (8.0%), exudation in 11 patients (5.5%), eczematous plaques in 3 patients (1.5%), pigmentation in 2 patients (1.0%) and depigmentation in 2 patients (1.0%).

In 225 patients whose skin lesion locations were identified, the lesions were most commonly present on the hands and feet and limbs (187 patients, 83.1%), followed by the perioral area (171 patients, 76.0%) and the perianal area (163 patients, 72.4%). Other locations included the perineum (133 patients, 59.1%), cheeks (91 patients, 40.4%), eye corners (54 patients, 24.0%), trunk (50 patients, 22.2%), auricles (29 patients, 12.9%), and occipital region (27 patients, 12.0%). Among the 80 patients for whom the sequence of lesion appearance was recorded, the lesions initially appeared in the perianal area in 28 patients (35.0%), the hands and feet and limbs in 19 patients (23.8%), the perioral area in 12 patients (15.0%), the cheeks in 9 patients (11.3%), the perineum in 7 patients (8.8%), the occipital region in 2 patients (2.5%), the trunk in 2 patients (2.5%), and the auricles in 1 patient (1.3%).

### Dermatologic tests

Skin swab culture was performed in 54 out of 231 patients (23.4%), with 35 patients (64.8%) showing the growth of various bacterial and/or fungal organisms. Among these, single bacterial infection was the most common, observed in 17 patients (48.6%), followed by mixed bacterial infections in 8 patients (22.9%), bacterial and fungal coinfections in 6 patients (17.1%), and single fungal infection in 4 patients (11.4%). Among the 35 patients, *Staphylococcus aureus* was the most common pathogen, identified in 18 patients (51.4%), followed by *Candida albicans* in 8 patients (22.9%).

Skin biopsy was performed in 61 (26.4%) patients. A histopathological examination of biopsy samples supported the diagnosis of AE in 55 patients (90.2%); this examination typically revealed necrolysis in the upper part of the epidermis.

### Laboratory abnormalities

Among 191 patients, 145 (75.9%) had zinc deficiency, with the lowest zinc level in the blood reported as 0.8 μg/dL (124) and the average level reported as 31.2 μg/dL. Among the 63 patients with reported causes of zinc deficiency, 37 cases (58.7%) were attributed to insufficient zinc intake, which included 24 cases (64.9%) related to low breast milk zinc levels. Additionally, 38 cases (60.3%) were attributed to impaired absorption, and 10 cases (15.9%) were attributed to increased demand. Furthermore, 13 patients (20.6%) presented with two or more contributing factors. Among the 87 patients whose ALP levels were recorded, 40 patients (46.0%) exhibited decreased ALP levels, with a minimum level of 15 U/L (94) and an average level of 74.3 U/L. Among the 55 patients with albumin measurements, 33 patients (60.0%) had reduced albumin levels, with a minimum level of 1.2 g/dL (70, 182, 184) and an average level of 2.0 g/dL. Hemoglobin levels were recorded in 63 patients, of whom 39 (61.9%) had decreased hemoglobin levels, with a minimum level of 5.5 g/dL (117) and an average level of 9.2 g/dL. Genetic information was identified in only 34 out of 231 cases (of which 156 were reported after the contribution of the *SLC39A4* gene was established), and mutations in the *SLC39A4* gene were confirmed in 32 of these individuals.

### Associated conditions

Fifty-seven patients (24.7%) (8, 18, 19, 21, 23, 26, 35, 37, 44, 47, 56, 57, 70, 87, 91, 101, 102, 104, 106–108, 112, 117, 125, 134, 137, 138, 145, 148, 151, 165, 167, 169, 172, 179, 181, 182, 184, 186, 193) were diagnosed with metabolic diseases: cystic fibrosis (CF) was present in 18 patients (31.6%), followed by maple syrup urine disease (MSUD) in 15 patients (26.3%), methylmalonic acidemia (MMA) in 7 patients (12.3%), ornithine transcarbamylase deficiency (OTC) in 4 patients (7.0%), and propionic acidemia (PA) in 4 patients (7.0%). Additionally, phenylketonuria (PKU) was diagnosed in 3 patients (5.3%), whereas biotinidase deficiency, Hartnup disease, d < 1.063 lipoprotein deficiency, nonketotic hyperglycinemia, aminoacidemia, and type 1 glutaric acidemia were each diagnosed in 1 patient (1.8%). Among those with metabolic diseases, 11 patients (19.3%) had zinc deficiency, and 7 of these patients (63.6%) had CF. Additionally, 29 patients had normal zinc levels, while 17 patients did not have their zinc levels tested.

Twenty-eight patients (12.1%) (11, 27, 33, 36, 43, 49, 53, 66, 80, 86, 96, 100, 107, 109, 121, 123, 129, 152, 158, 159, 163, 168–171, 192, 195, 198) were documented as preterm, of whom 26 (92.9%) had zinc deficiency.

Twenty patients (8.7%) (39, 45–47, 50, 79, 90, 92, 140, 153, 166, 169, 175, 181, 184, 185, 191, 198) were diagnosed with gastrointestinal diseases, among whom 7 (35.0%) had received total parenteral nutrition. Zinc levels were not recorded in 1 patient (169), but zinc deficiency was observed in all of the remaining 19 patients (95.0%). No patients were found to have liver or kidney diseases.

Furthermore, malnutrition was identified in 9 patients (3.9%) (11, 32, 39, 65, 84, 87, 102, 127, 128), including 7 patients (77.8%) presenting with diarrhea, 1 patient (11.1%) who was diagnosed with MSUD (87), and 1 patient (11.1%) who was a preterm infant born at 31 weeks gestation (11). Zinc deficiency was observed in 6 of the malnourished patients (66.7%).

### Treatment and prognosis

Among the 231 patients, 174 (75.3%) received zinc supplementation, including 137 patients (78.7%) with zinc deficiency, 25 patients (14.4%) with normal zinc levels, and 12 patients (6.9%) with unrecorded zinc levels; of the patients receiving zinc supplementation, 159 (91.4%) demonstrated therapeutic efficacy. Among the 25 patients with normal zinc levels who received supplementation, 21 patients (84.0%) responded effectively, whereas 4 patients (16.0%) had an ineffective response, all of whom also had concomitant metabolic disorders (35, 37, 44, 145).

Among the 174 patients who received zinc supplementation, 4 patients (2.3%) were treated with a zinc dose of < 1 mg/kg/day, 55 patients (31.6%) received a dose of ≥ 1 to ≤ 3 mg/kg/day, 20 patients (11.5%) received a dose of > 3 to ≤ 5 mg/kg/day, and 10 patients (5.8%) were treated with a dose of > 5 mg/kg/day (Table 3). In 85 patients (48.9%), either the zinc dose was not weight-adjusted or the dosage was not recorded. With respect to rash recovery time, 46 patients (26.4%) experienced resolution of the rash within 1 week, 50 patients (28.7%) recovered within 1–4 weeks, and 22 patients (12.6%) experienced resolution of the rash after more than 4 weeks. In 41 patients (23.6%), zinc supplementation was effective, but the time to rash resolution was not specified. For 5 patients (2.9%), the outcome was unknown. Zinc supplementation was ineffective in 10 patients (5.8%), all of whom had metabolic disorders, including CF, PA, MMA, MUSD, nonketotic hyperglycinemia and aminoacidemia. We compared the effects of different doses of zinc supplementation on lesion recovery time, as shown in the chi-square analysis in Table 2, and found that different dosages of zinc had no significant effect on lesion recovery time (*χ*^2^ = 2.880, *p* = 0.824).

Thirty out of 32 (93.8%) patients with *SLC39A4* gene mutations received zinc supplementation, with 29 patients (96.7%) showing improvement (12, 15, 25, 29, 61, 65, 68, 71, 73, 75, 77, 80, 95, 98, 110, 118, 120, 130, 135, 143, 156, 173, 187–189, 194). Only one patient did not respond to zinc treatment and subsequently died (164). Among these patients, 12 (40.0%) were treated with a zinc dose of ≥ 1 to ≤ 3 mg/kg/day, whereas 6 patients (20.0%) received a dose of > 3 mg/kg/day. In 12 patients (40.0%), the zinc dosage was either not weight-adjusted or was not recorded. Among the 29 patients who recovered, 12 (41.4%) achieved resolution within 1–4 weeks, 5 patients (17.2%) experienced resolution after more than 4 weeks, and the resolution time was unspecified for 12 patients (41.4%).

Among the 57 patients with metabolic diseases, 53 received treatment, 21 of whom received zinc supplementation. Among these patients, only 2 patients (9.5%) improved (47, 169), 5 patients (23.8%) experienced ineffective zinc supplementation and subsequently died from sepsis or metabolic decompensation (37, 104, 112, 125), 5 patients (23.8%) recovered after correcting metabolic disorders following ineffective zinc treatment (35, 44, 117, 134, 145), and 9 patients (42.9%) recovered with a combination of zinc supplementation and correction of metabolic imbalances (8, 18, 47, 57, 107, 108, 167, 181, 186). In contrast, 32 patients were solely treated for metabolic imbalances, and all (100%) recovered (19, 21, 23, 26, 37, 56, 70, 87, 91, 104, 106, 137, 138, 148, 151, 172, 179, 182).

All 26 (100%) premature infants with zinc deficiency improved after zinc supplementation. Among patients with gastrointestinal diseases, 18 (90%) received zinc supplementation, and all (100%) demonstrated improvement (39, 45, 47, 50, 79, 90, 92, 140, 153, 166, 169, 175, 181, 185, 191, 198). Three malnourished patients who received zinc supplementation (100.0%) showed improvement. Similarly, all patients (100%) with zinc deficiency due to low breast milk zinc levels improved after zinc supplementation (27, 28, 33, 42, 49, 96, 98, 103, 110, 118, 121, 126, 129, 133, 154, 158, 160, 170, 176, 178, 183, 198).

**Figure 2:**
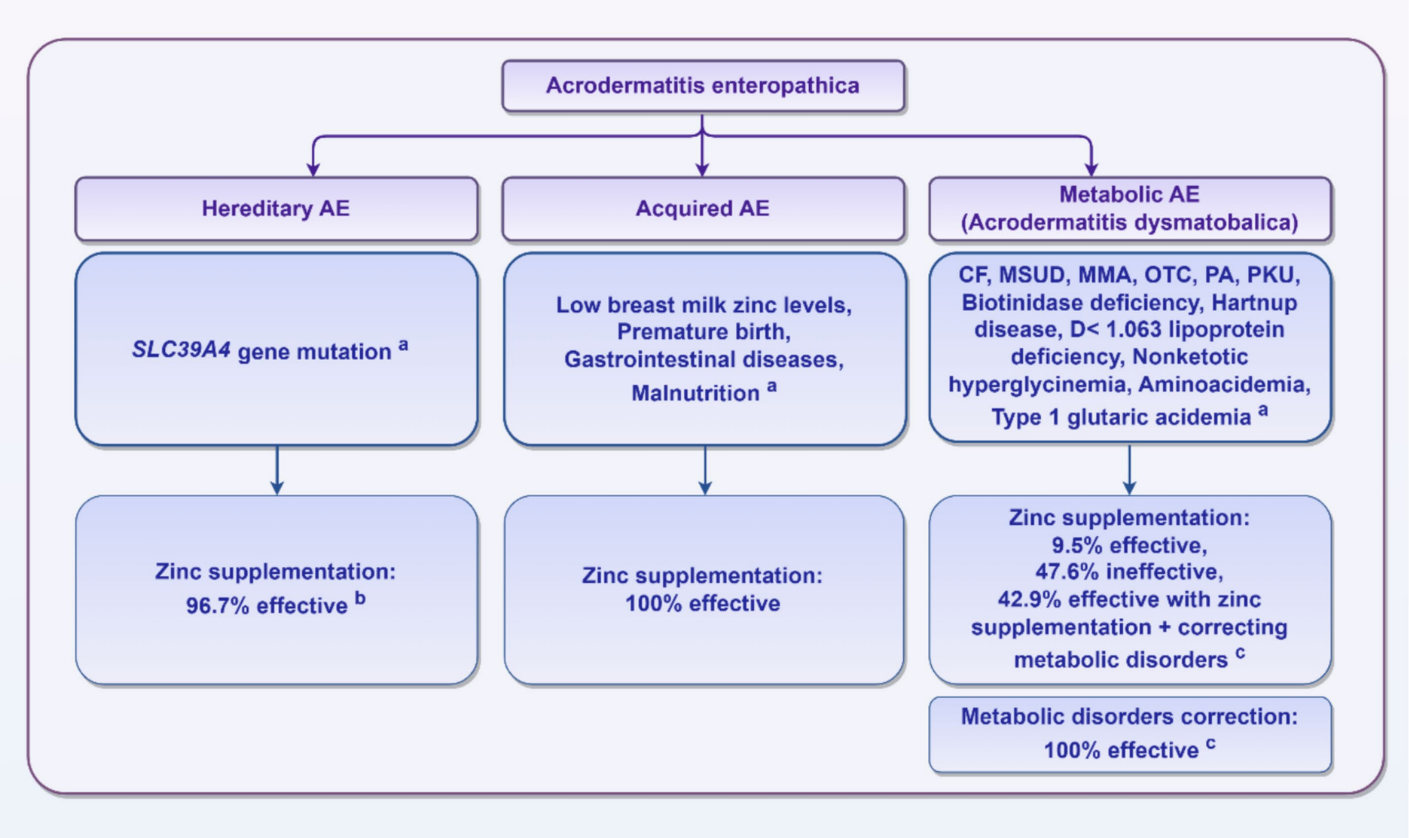
AE classification and treatment effects. ^a^These reasons are limited to the specific reasons mentioned in the published literature summarized in this review and do not include unreported cases. ^b^Thirty patients with *SLC39A4* gene mutations received zinc supplementation, with 29 patients (96.7%) showing improvement; one patient did not respond to zinc treatment and subsequently died. ^c^Among the 57 patients with metabolic diseases, 53 patients received treatment, including 21 patients who received zinc supplementation. Among these, only 2 patients (9.5%) showed improvement, 10 patients (47.6%) had an ineffective treatment response, and 9 patients (42.9%) recovered with a combination of zinc supplementation and correction of metabolic imbalances. In contrast, 32 patients were solely treated for metabolic imbalances, and all (100%) recovered.

In summary, there are significant differences in treatment methods, efficacy, and prognosis for AE based on their various etiologies. Figure 2 provides a brief summary of this information.

## Discussion

The diagnosis of AE relies on clinical dermatological characteristics, with the presence of zinc deficiency often supporting the clinical diagnosis (10). According to our analysis, zinc deficiency was observed in only 75.9% of cases, which is consistent with previously reported findings (199). Our results indicate that the two major causes of zinc deficiency are insufficient intake and impaired absorption. Zinc deficiency occurs when the zinc supply fails to meet the physiological needs of children. Among the various causes, low zinc levels in breast milk are particularly common, which may be partly attributed to mutations in the *ZNT2* gene, which is responsible for supplying human milk with zinc (200–202). Furthermore, zinc absorption disorders are prevalent in genetic AE and various gastrointestinal diseases, where damage to intestinal epithelial cells and their barrier leads to impaired zinc absorption. The patients in our review included those with gastrointestinal conditions such as intestinal immaturity in preterm infants, celiac disease, infantile refractory diarrhea, neonatal necrotizing enterocolitis (NEC), food allergies, and postintestinal resection (25, 27, 29, 36, 39, 46, 47, 65, 68, 71, 100, 159, 166).

Consistent with previous reports (203), the triad of dermatitis, diarrhea, and alopecia simultaneously appeared in 21.1% of patients. In addition, neuropsychiatric manifestations (33.5%) were the most prominent complications of AE, with growth restrictions observed in more than 20% of patients. Remarkably, 13.4% of patients presented with dermatitis as the only symptom of AE. After analyzing the characteristics of these 28 patients, we found that patients with only skin symptoms were all first-episode AE patients (8, 12, 25, 30, 31, 37, 59, 86, 89, 97, 103, 104, 126, 129, 134, 137, 138, 159, 163, 165, 175–177, 198), and the time from the onset of symptoms to diagnosis was relatively short (3.8 weeks, range: 0.7–8 weeks). These findings suggest that AE should be considered even in the absence of the full triad, particularly in first-onset cases. Early diagnosis and treatment can help avoid adverse complications and improve patient prognosis, emphasizing the need for clinician vigilance.

Currently, zinc supplementation remains the primary treatment approach for AE. Based on our data, 174 AE patients received zinc supplementation at varying dosages, with 159 (91.4%) demonstrating therapeutic efficacy. Interestingly, this group also included 21 patients (13.2%) who had normal zinc levels, suggesting that zinc levels in the blood should not be the sole indicator of zinc deficiency. Patients with normal zinc blood levels may still benefit from zinc supplementation, and a comprehensive assessment should consider the patient’s response to zinc supplementation as well. In terms of dosage distribution, when cases with unclear zinc supplementation doses were excluded, the most commonly used dosage for AE, including hereditary AE, was 1–3 mg/kg/day, which was administered in 61.8% (55/89) of the patients receiving supplementation. Table 2 further confirms the efficacy and appropriateness of the 1–3 mg/kg/day dosage in AE treatment. With respect to lesion recovery time, after excluding patients with unrecorded recovery times and unrecorded therapeutic efficacy, the majority of patients (81.4%, 96/118) recovered within 4 weeks, with nearly half of the patients (47.9%, 46/96) recovering within 1 week. However, no patients with hereditary AE were observed to recover within 1 week after zinc supplementation, suggesting that it may take more time to replenish the zinc transport pool. In summary, zinc supplementation has shown significant efficacy, with 1–3 mg/kg/day reported as the most common dosage; however, individualized dose adjustment is necessary, particularly for the long-term management of patients with hereditary AE.

As mentioned earlier, reports have indicated that metabolic disorders can mimic the manifestations of AE, a condition that Tabanhoglu and colleagues referred to as acrodermatitis dysmetabolica (19). Consistent with these findings, our analysis of 57 cases of AE associated with metabolic disorders revealed that the majority of cases occurring during the decompensation of metabolic diseases exhibited improvement following targeted nutrient supplementation. These metabolic diseases include, but are not limited to, CF, MSUD, MMA, OTC, PA, and PKU. In fact, the most important aspect of managing these metabolic diseases is dietary control, particularly the use of specific formulas during early life. However, if monitoring is insufficient, it can easily result in deficiencies in amino acids or lead to malnutrition. Some factors such as pancreatic enzymes (204) and albumin (205, 206) have been reported to affect zinc absorption and bioavailability, and zinc ionophores such as amino acids and peptides have been documented to enhance zinc absorption (207). Furthermore, some essential amino acids, such as isoleucine and valine, are essential for keratinocyte metabolism (208). As shown in the results, most patients with AE and metabolic disorders recovered by solely addressing their metabolic imbalances, which included supplementation with essential amino acids or pancreatic enzymes. Additionally, a few patients only showed improvement after supplementing with pancreatic enzymes or amino acids when zinc supplementation was ineffective. Therefore, it is reasonable to hypothesize that certain amino acid metabolic abnormalities that occur during the inadequate management of metabolic diseases contribute directly to AE. However, at present, the molecular mechanisms of nonhereditary AE have not been sufficiently studied, particularly AE secondary to metabolic disorders. In the future, research can further explore the relationship between metabolic disorders, zinc metabolism, and AE manifestations.

In summary, AE is classified into three types: hereditary, acquired, and metabolic AE (Acrodermatitis dysmetabolica). Hereditary AE results from inherited zinc malabsorption, often due to mutations in the *SLC39A4* gene. It responds well to zinc supplementation, which usually requires lifelong treatment. Acquired AE is caused by low zinc intake, which may result from factors like low zinc levels in breast milk, prematurity, gastrointestinal diseases, or malnutrition. It also responds to zinc supplementation. Metabolic AE occurs alongside metabolic comorbidities, treatment may focus solely on correcting metabolic imbalances, or it may include zinc supplementation. Clinicians should be vigilant to identify AE subtype early and provide proper treatment to avoid adverse complications and improve patient prognosis. During the clinical diagnosis and treatment process, physicians should develop a reasonable therapeutic strategy by integrating a comprehensive patient history and a methodological collaborative approach, including laboratory tests, screening for genetic metabolic disorders, and genetic analysis.

## Limitations

This literature review has several limitations, including publication bias and the predominance of case reports. Additionally, there is limited information regarding *SLC39A4* mutations, making it difficult to analyze the incidence of hereditary AE. Furthermore, there is insufficient quantitative data for statistical analysis, particularly concerning different dosages and recovery times. Another limitation is that given the increased understanding of metabolic diseases in recent years, several metabolic disorders related to AE symptoms have been identified. While this review aims to include such patients as comprehensively as possible, it is possible that some cases may not have been captured.

## Data Availability

The raw data supporting the conclusions of this article will be made available by the authors, without undue reservation.
